# Evaluating a virtual reality–delivered mindfulness intervention for anxiety: a mixed-methods study in real-world community and school settings

**DOI:** 10.3389/fpsyt.2025.1669287

**Published:** 2025-12-11

**Authors:** Sima Rafiei, Kristof Santa, Nicola Honey, David Tully, Barbara Mezes

**Affiliations:** 1 University of Liverpool Faculty of Health and Life Sciences, Liverpool, United Kingdom; 2 Department of Psychology, Manchester Metropolitan University, Manchester, United Kingdom; 3 Scenegraph Studios, Birkenhead, United Kingdom

**Keywords:** adolescent anxiety, virtual reality, mindfulness, digital mental health, GAD-7, school-based intervention, mixed methods

## Abstract

**Introduction:**

Adolescent anxiety is rising globally, yet access to engaging mental health support remains limited, especially outside clinical care. Virtual reality (VR) offers a promising platform for delivering immersive mindfulness experiences. This study evaluated *SpiritVR Journey*, a six-session VR mindfulness programme designed for young people, delivered in real-world school and community environments.

**Methods:**

Using an exploratory pre–post mixed-methods design, data were collected from 53 participants across schools, community organisations, and youth mental health services. Recruitment was facilitator-led and internal to each site. Quantitative data were gathered before and after each session using an adapted Generalised Anxiety Disorder scale (GAD-7), while qualitative reflections were obtained from participant diary entries. Sessions were delivered approximately once per week, depending on site scheduling.

**Results:**

Participants showed a significant reduction in self-reported anxiety symptoms across sessions (mean GAD-7 change = 4.88; Cohen’s d = 2.06). Qualitative themes highlighted relaxation, emotional relief, and improved self-regulation. The immersive format appeared particularly engaging for individuals who had found traditional mindfulness approaches challenging.

**Discussion:**

Findings provide preliminary evidence of impact and acceptability of immersive VR mindfulness in youth and community contexts. While results suggest promise for future scalable applications, broader feasibility depends on access to VR hardware, training of facilitators, and setting-specific implementation support.

## 1 Introduction

Virtual reality (VR) refers to a computer-generated, interactive three-dimensional environment that users can explore and manipulate in real time, creating an immersive sense of presence (1). Advances in VR technology have expanded its applications far beyond entertainment, extending into healthcare, education, and psychological research (2). Within mental health, VR has become a versatile tool to simulate real-world situations in safe, controlled contexts, supporting exposure therapy, cognitive-behavioural interventions, and skills training. For example, VR exposure therapy (VRET) has been shown effective in treating specific phobias such as fear of flying and fear of heights, enabling patients to face anxiety-provoking stimuli without leaving the clinical setting (3, 4). VR has also been used in other psychiatric conditions, including obsessive–compulsive disorder, where early work showed promising effects (5).

### 1.1 VR for anxiety and personalisation

Anxiety disorders have been the most frequent focus of VR applications. Evidence indicates that virtual reality exposure therapy is effective in treating specific phobias, social anxiety, and post-traumatic stress disorder (6). A meta-analysis of randomised controlled trials demonstrated that VR-based interventions can achieve outcomes comparable to, and in some cases exceeding, those of traditional *in vivo* exposure (7). A distinctive advantage of VR lies in its ability to provide repeatable, ecologically valid, and potentially adaptable environments, which can enhance engagement, reduce costs, and support broader scalability (8). Current health policy increasingly highlights the importance of personalisation in mental health interventions (9). Although Spirit VR Journey was delivered as a structured six-session programme, VR technology offers inherent possibilities for tailoring such as allowing users to adjust the pace of sessions, select preferred environments, or modulate intensity.

### 1.2 Mindfulness-based VR interventions

In addition to its established role in exposure therapy, VR is increasingly being applied to mindfulness, relaxation, and emotion-regulation interventions. Mindfulness-based interventions (MBIs), which encourage present-moment awareness and non-judgmental acceptance, are well supported in the literature as effective strategies for reducing symptoms of anxiety, depression, and stress (10, 11). Delivering mindfulness through VR offers distinctive advantages: immersive, calming environments can minimise distraction, foster attentional focus, and support experiential learning in ways that may be more engaging than traditional formats (12, 13). Studies have reported that VR-enhanced mindfulness can alleviate anxiety and stress, strengthen emotional regulation, and improve adherence relative to non-immersive or audio-based practices (14; 15–17).

### 1.3 Theoretical and empirical background

The theoretical foundation for this study is informed by the Mindfulness-to-Meaning Theory (18), which proposes that mindfulness training facilitates positive reappraisal of stressors, enabling individuals to reinterpret distressing experiences in less threatening ways. Delivering mindfulness through VR may amplify these effects by providing immersive, distraction-free environments that heighten attentional focus, deepen experiential learning, and promote a stronger sense of presence (12, 18, 19).

Despite these theoretical advantages, empirical research on VR-delivered mindfulness remains at an early stage. Systematic reviews highlight that most studies are constrained by small sample sizes, single-session protocols, and controlled laboratory settings, limiting generalisability and making it difficult to determine optimal “dosage” or sustained effects (12, 16). More recent empirical trials suggest multi-session interventions may reduce anxiety and improve emotional regulation, but studies are still relatively few and often short in duration (17). Furthermore, research has focused largely on clinical or convenience samples, with fewer studies conducted in naturalistic settings or broader young adult populations where transdiagnostic approaches may have wider public health impact (20).

Together, these gaps underscore the need for larger, multi-session, ecologically valid evaluations of VR mindfulness programs that not only assess symptom change but also explore participants’ lived experiences and engagement.

### 1.4 Related work

A growing body of research has examined the application of VR to mindfulness-based interventions (MBIs). Systematic reviews indicate that VR mindfulness consistently reduces stress, anxiety, and negative affect, while enhancing engagement and presence compared to non-immersive or audio-guided formats (12, 16). However, most existing studies are limited by small samples, single-session protocols, and controlled laboratory settings, restricting conclusions about long-term effectiveness and real-world feasibility (12, 16).

### 1.5 Limitations of VR interventions and study aims

Although the use of VR in mental health care is expanding, several challenges remain. Practical barriers include the cost of headsets, the availability of suitable hardware, and the fact that not all potential users are equally comfortable with the technology. Moreover, VR applications can produce side effects such as cybersickness or visual fatigue, which may discourage uptake and limit the duration of use (21). Another important issue is that most VR mindfulness studies to date have focused on immediate symptom change; far less is known about whether benefits persist once the intervention ends. This lack of long-term evidence continues to constrain claims about durability and wider scalability.

In light of these considerations, our study set out to examine the short- to medium-term effects of *Spirit VR Journey*, a structured six-session mindfulness programme delivered in VR. The primary goal was to establish whether such an intervention could lead to clinically meaningful reductions in anxiety symptoms and to capture participants’ experiences of engaging with mindfulness in a virtual environment. Questions of sustained benefit over the longer-term fall beyond the scope of this evaluation but represent an important direction for future research.

### 1.6 The purpose of the study

This study extends earlier work by examining *Spirit VR Journey*, a structured six-session mindfulness programme delivered in virtual reality and implemented in a naturalistic setting with young adults. In contrast to many previous trials, we incorporated both quantitative assessment of anxiety symptoms (GAD-7) (22) and qualitative accounts of participant experience. This mixed-methods approach was intended not only to test the clinical effectiveness of the programme but also to shed light on how users engaged with mindfulness in a virtual environment. By tracking outcomes across multiple sessions, we were able to examine patterns of change over time and to identify points at which therapeutic benefits may be most pronounced. The inclusion of qualitative feedback provided additional insight into mechanisms of change and into the feasibility and acceptability of VR-based mindfulness in everyday contexts.

## 2 Methods

### 2.1 Study design and participants

This study employed an exploratory pre–post mixed-methods design using a secondary analysis of data collected during the real-world deployment of *SpiritVR Journey*, a six-session virtual-reality (VR) mindfulness intervention aimed at reducing anxiety symptoms. The term *secondary* denotes that the present research team conducted an independent academic analysis of anonymised data originally collected by Scenegraph Studios Ltd. within the Innovate UK–funded programme *Mindset: Extended Reality for Digital Mental Health* (23, Strand1; GrantNo.10053224). The original, industry-led evaluation focused primarily on technical feasibility and user-experience metrics, including headset usability, comfort, and engagement patterns and remains unpublished while subsequent project phases are ongoing. The current analysis, undertaken in collaboration with the University of Liverpool, extended this work by examining short-term psychological outcomes and participant reflections to generate preliminary, hypothesis-building evidence on anxiety reduction and user experience.

The recruitment process aimed to achieve a heterogeneous valid sample across diverse real-world contexts, including schools, community organisations, and mental health services. In one of our recruitment site at a school, pastoral-care staff identified students experiencing moderate anxiety or stress and invited them to participate during wellbeing sessions. In two community recruitment sites, participants were enrolled to integrate VR-based wellbeing initiatives into their youth mentoring programmes. Recruitment within mental health services took place through relevant mental health services. Across all sites, 53 provided informed consent and completed at least one VR session. Recruitment numbers varied between sites, primarily due to the limited availability of VR headsets and scheduling constraints inherent to each organisational setting.

Participants were eligible for inclusion if they were adolescents or young adults (approximately 13–25 years) identified by site facilitators as experiencing mild to moderate anxiety or general wellbeing concerns, were willing to participate in a multi-session VR mindfulness programme, and had the capacity to provide informed consent (or parental or guardian consent for minors). Exclusion criteria included individuals experiencing an acute mental-health crisis requiring specialist or emergency care, those with known epilepsy, vestibular disorders, or severe motion sickness triggered by VR exposure, and anyone unable to tolerate or physically wear a VR headset. Eligibility was assessed informally by facilitators through routine safeguarding and wellbeing checks conducted within each organisation, rather than through standardised psychometric tools, to maintain the ecological authenticity of the real-world recruitment process. Four prospective participants were excluded due to motion sensitivity or headset intolerance, and no other exclusions were reported.

The final sample comprised 53 participants (32% female, 68% male). Six participants were under the age of 18 (all female), while the remaining participants were 18 years or older. Demographic information, including age, gender, and recruitment site, was recorded anonymously. One participant discontinued participation after the first session, reporting discomfort with VR, whereas all others completed between one and six sessions depending on local scheduling and organisational capacity.

No *a priori* sample size calculation was conducted, as the study was exploratory in nature and based on pre-existing data from a feasibility deployment. The inclusion of varied settings: school, community, and clinical was intentional, designed to explore the acceptability and applicability of the intervention across real-world contexts rather than to permit statistical comparisons between sites. Although group sizes were relatively small, this variation reflects the ecological implementation conditions and enhances the external validity of the findings.

Descriptive comparisons of mean GAD-7 (22) change scores showed no systematic differences between individual and group delivery formats, suggesting minimal bias related to delivery mode. Sessions were delivered approximately once per week, with timing adjusted according to school timetables or community-centre availability. While some variability in delivery frequency occurred, exploratory analyses indicated that these differences did not correlate with changes in anxiety outcomes.

As the study did not include a control arm, findings should be interpreted as preliminary and hypothesis-generating, providing early evidence of potential benefit rather than causal inference.

### 2.2 Intervention

*SpiritVR Journey* is a self-guided, six-session virtual reality (VR) mindfulness programme designed to cultivate relaxation, present-moment awareness, and emotional regulation. The content was co-developed with clinical psychologists, wellbeing practitioners, and psychotherapists, following an iterative, user-centred design process that involved adolescents and young adults in pilot workshops conducted in both school and community settings. Feedback from these workshops on usability, comfort, and relevance informed multiple refinements to optimise engagement and accessibility for younger users.

Several adaptations were made to tailor the programme from traditional adult mindfulness protocols. Session durations were reduced to 15–20 minutes to align with adolescent attention spans, and guided narration employed accessible, non-technical language. Visual metaphors, such as floating orbs to represent thoughts and a mountain symbolising resilience, were incorporated to help concretise abstract mindfulness concepts. Interactive breathing exercises were embedded to encourage active engagement throughout the sessions.

The programme was developed using Unreal Engine and deployed on Meta Quest 2 headsets. Design principles prioritised ease of use and accessibility, with minimal navigation demands to accommodate first-time VR users, including those with neurodiverse profiles. All sessions were facilitated in supervised environments to ensure participant safety and provide technical support. Each of the six sessions focused on a specific mindfulness skill: mindful sensing of environmental stimuli, anchored breath awareness, somatic awareness through body scanning, observing thoughts as visualised orbs and releasing them, mountain meditation to promote stability and resilience, and loving-kindness meditation to foster compassion toward self and others. Each session began with a paced breathing exercise and concluded with a guided transition back to the physical environment to minimise disorientation.

Variability in delivery frequency (approximately once per week) and format (individual or small-group sessions) reflected the pragmatic realities of real-world implementation. Although the study was not powered to compare formats formally, exploratory analyses indicated comparable pre-to-post reductions in anxiety across delivery modes, suggesting that these contextual variations did not introduce systematic bias into the findings.

### 2.3 Measures

#### 2.3.1 Quantitative measures

Anxiety symptoms were measured using a modified version of the Generalized Anxiety Disorder-7 (GAD-7) scale, administered immediately before and after each VR session. The validated GAD-7 typically assesses symptoms over the past two weeks; however, this recall period is unsuitable for detecting changes within the ~20-minute intervals of the present study. To enhance ecological validity, the instructions (rather than item content) were adapted to assess participants’ current state (“right now/at this moment”) instead of a two-week recall period, with response options indicating immediate severity (“Not at all,” “Mildly,” “Moderately,” “Severely”). Such adaptations are widely used in methodologies that collect momentary data, such as Experience Sampling Method (ESM), where the focus is on capturing in-the-moment states while maintaining conceptual alignment with established trait measures (24, 25). Although the GAD-7 was originally validated for a two-week recall period, adaptations of the scale and similar anxiety measures for shorter intervals have been reported in intervention and ESM studies, supporting its use here for session-level symptom tracking (24–28). All responses were self-reported using the VR platform’s integrated survey interface and recorded digitally in real time, ensuring consistent administration across participants and sessions. For the overall pre- to post-programme comparison, a clinically meaningful change was defined as a decrease of ≥4 points on the standard GAD-7 scale, consistent with established benchmarks, and applied only to baseline-to-final-session outcomes rather than to immediate session-level changes (29). The adapted version of the GAD-7 used in this study, including modified instructions and response options, is provided in **Supplementary Material 1**.

#### 2.3.2 Qualitative measures

Qualitative data were drawn from participant diary entries completed immediately after each VR session. These diaries captured open-ended reflections on participants’ emotional states, bodily sensations, cognitive responses, and perceived impact of the VR environment on anxiety and wellbeing.

### 2.4 Procedures

Following informed consent, which was obtained verbally or in writing depending on the site and stage of data collection, participants completed the adapted GAD-7 immediately before and after each session. After each session, participants were invited to complete a diary entry documenting their experience. Sessions were delivered either individually or in small groups depending on site resources. This contextual detail was not recorded systematically at the participant level, although the intervention content and delivery within the headset were identical across formats. No financial incentives were provided for participation. The study received ethical approval from the University of Liverpool.

### 2.5 Data analysis

Quantitative analyses were performed using Stata 18. Descriptive statistics were used to summarise demographic characteristics and anxiety scores across sessions. Paired t-tests assessed pre- and post-intervention changes in GAD-7 scores, with effect sizes calculated using Cohen’s d. The proportion of participants achieving a clinically meaningful improvement (≥4-point reduction) between baseline and final session was also calculated. Repeated measures analysis of variance (ANOVA) was used to examine trends in anxiety scores across multiple sessions. Estimated marginal means (‘predicted’ scores) were derived from the regression model to provide adjusted averages of GAD-7 scores before and after each session, with standard errors and confidence intervals reflecting model-based estimates rather than raw means.

Qualitative data were analysed using inductive content analysis. This involved open coding to identify emergent concepts, axial coding to explore relationships between themes, and selective coding to integrate findings into explanatory categories. NVivo software was used to support data management and coding.

Findings from quantitative and qualitative analyses were triangulated to provide a richer understanding of the intervention’s acceptability, patterns of engagement, and short-term impact on anxiety. The absence of a control group is acknowledged as a limitation of the design, and results are interpreted as preliminary and hypothesis-generating rather than definitive evidence of causal impact.

## 3 Results

### 3.1 Participants’ characteristics

A total of 53 participants contributed data to the study. Of these, 17 (32.1%) identified as female and 36 (67.9%) as male. Age information was available for a subset of participants, with nine participants (17%) aged under 18 years and one participant (1.9%) aged 18 years or older; the remaining participants did not report their age. Recruitment sites included schools (n = 6), mental health services (n = 32), and other or unspecified community-based settings (n = 15). No participants recruited through the mental health organisations declined to participate. Within the school setting, one participant attended a single session but chose not to continue, stating that “VR wasn’t for her.” All other participants completed at least one session, with some completing all six sessions depending on the delivery model at their site. This distribution reflects the diverse educational, clinical, and community contexts in which the intervention was implemented.

All 53 participants who met inclusion criteria provided informed consent prior to participation. Of these, 45 provided written consent and 8 provided verbal consent via their facilitators, in accordance with the procedures approved by the University of Liverpool ethics committee. For the six participants under 18 years of age, parental consent forms were obtained through the participating school.

### 3.2 Overall anxiety reduction (pre- to post-intervention)

Participants’ predicted GAD-7 score (i.e., the model-estimated average across sessions) immediately before a session was 8.30 (SE = 0.48), 95% CI [7.36, 9.25], which differs significantly from zero (z = 17.22, p <.001) The estimated marginal means for pre- and post-session anxiety scores are presented in **Table 1**. Immediately after a session, the predicted score dropped to 3.42 (SE = 0.48), 95% CI [2.47, 4.36], also highly significant (z = 7.09, p <.001).

These margins confirm a robust average reduction of 4.88 points in GAD-7 from pre- to post-session. This quantifies the large, immediate effect of each VR intervention session on reducing anxiety. A large effect size of Cohen’s d = 2.06 means that the average post-intervention anxiety score is 2.06 standard deviations lower than the pre-intervention score.

**Table 1:** The estimated marginal (predicted) GAD-7 scores over all six sessions, for pre- and post-intervention.

Time	Margin/mean	Std. Err	z	p-value	95% CI
Pre	8.30	0.48	17.22	<.001	[7.35, 9.24]
Post	3.41	0.48	7.09	<.001	[2.47, 4.36]
Cohen’s d =2.06					

### 3.3 Pre-intervention anxiety levels

A one-way ANOVA was conducted to examine whether participants’ baseline GAD-7 anxiety scores (measured immediately before each session) differed significantly across the six SpiritVR sessions. The one-way ANOVA results comparing baseline anxiety across sessions are shown in **Table 2**. This analysis addressed only pre-session (baseline) scores and did not examine the magnitude of change between sessions. No significant differences were observed in baseline (pre-intervention) anxiety scores across the six lessons (F (5,142) = 1.34, p = .25), indicating that participants entered each session with comparable levels of anxiety.

**Table 2:** Analysis of variance (ANOVA) comparing pre-intervention anxiety scores across six VR sessions.

Source	Partial SS	df	MS	F	Prob > F
Model	84.65	5	16.93	1.34	0.25
Lessons	84.65	5	16.93	1.34	0.25
Residual	1796.99	142	12.65	Not applicable	Not applicable
Total	1881.64	147	12.80	Not applicable	Not applicable

### 3.4 Within-session anxiety changes (session-by-session)

To assess immediate therapeutic effects, GAD-7 change scores (post-session minus pre-session) were computed for each session separately. In contrast to the baseline comparison reported above, these analyses examined session-by-session changes (pre–post differences). Session-specific changes in GAD-7 scores are summarised in **Table 3**. Although reductions were observed in all sessions, exploratory comparisons suggested that the first session was associated with the largest average decrease, while later sessions maintained these gains with smaller fluctuations. For example, the change score in Session 1 differed significantly from that in Session 6, despite baseline levels being comparable. Participants experienced significant anxiety reductions after each session, with mean change scores ranging from –4.56 to –5.39 across six lessons.

**Table 3:** Mean GAD-7 score change per session.

Lessons	Mean	SD	N	Min	Max
1	-5.39	2.18	27	-10	-2
2	-4.56	2.10	25	-10	-1
3	-4.84	3.26	25	-12	0
4	-4.92	2.12	24	-9	-1
5	-4.60	2.12	25	-8	-1
6	-4.95	2.38	22	-9	0
Total	-4.88	2.37	148	-12	0

**Table 3** presents session-by-session changes in GAD-7 scores across six VR intervention lessons. As we can see from the table, the largest benefit was observed after the first session, suggesting novelty or initial responsiveness may play a role. Subsequent sessions maintained the gains, with slight variations. Thus, it is evident that the intervention demonstrates a consistent within-session reduction in anxiety symptoms, with a mean GAD-7 change of -4.88, which is clinically meaningful. These results provide strong support for the immediate therapeutic benefit of each VR session. Full fixed-effect estimates from the linear mixed-effects model are presented in **Table 4**. 

**Table 4:** Fixed effects from linear mixed-effects model predicting GAD-7 scores across time (pre *vs*. post) and session (1–6).

Predictor	Coef. (SE)	z	p-value	95%CI
Intercept (Session 1, Pre)	9.21(0.60)	15.36	<.001	[8.03, 10.38]
Time: Post *vs* Pre	-5.39(0.62)	-8.46	<.001	[-6.61, -4.17]
Session (pre-session)				
Session 2	-0.55(0.64)	-0.85	0.393	[-1.80, 0.71]
Session 3	-1.25(0.65)	-1.92	0.055	[-2.52, 0.02]
Session 4	-1.05 (0.67)	-1.57	0.117	[-2.36, 0.26]
Session 5	-0.93(0.68)	-1.37	0.169	[-2.27, 0.40]]
Session 6	-1.85(0.73)	-2.54	0.011	[-3.27, -0.42]
Time × Session (Post *vs* Pre)				
× Session 2	0.83(0.90)	0.92	0.357	[-0.93, 2.59]
× Session 3	0.55 (0.9)	0.61	0.542	[-1.21, 2.31]
× Session 4	0.47(0.91)	0.52	0.604	[-1.31, 2.25]
× Session 5	0.79(0.90)	0.88	0.381	[-0.97, 2.55]
× Session 6	0.43(0.93)	0.47	0.641	[-1.39, 2.26]
Random‐effects variances				
Session slope (ID): Var = 0.0835 (SE = 0.057)				
Intercept (ID): Var = 4.2606 (SE = 1.527)				
Residual: Var = 5.2541 (SE = 0.478)				

Main effect of time: There was a highly significant pre-to-post reduction in anxiety, b = −5.39 (SE = 0.62), z = -8.64, p <.001, 95% CI [-6.61, -4.17]. On average, participants’ GAD-7 scores dropped by about 5.4 points per session.

Main effect of session: Pre-session anxiety showed a small downward trend over the six lessons; only Session 6 differed significantly from Session 1 (b = -1.85, p = .011), suggesting a modest decrease in baseline anxiety by the final lesson.

Time × Session interaction: None of the session-by-session interaction terms reached significance (all p ≥.357), indicating that the magnitude of the pre-to-post reduction was stable across lessons 1–6. These results confirm a robust immediate benefit of each VR mindfulness session (large, consistent reductions in GAD-7) with no evidence that this effect varied meaningfully from session to session.

**Figure 1:**
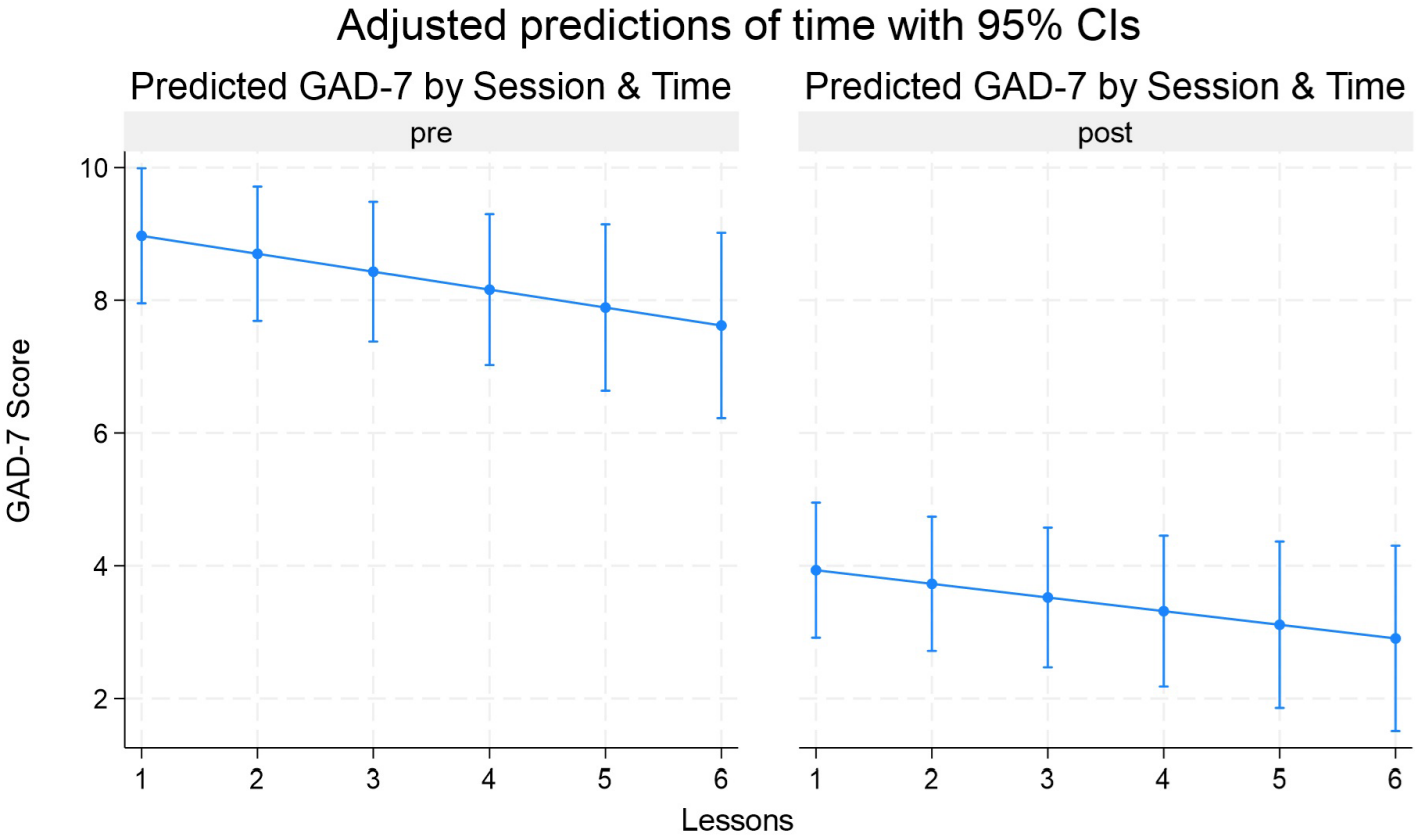
Adjusted predictions of GAD-7 by session and time.

**Figure 1** shows the model‐based predicted GAD-7 scores (with 95% confidence intervals) for each of the six lessons, separately for before (“pre”) and after (“post”) each VR session.

Pre-session panel (left): Baseline anxiety starts at about 9.0 points before Lesson 1 and then declines steadily to about 7.5 by Lesson 6. This gradual downward slope reflects the small but significant linear session effect in the mixed model (≈ −0.29 points per lesson, p=0.024), meaning participants came into later sessions with somewhat lower anxiety than they did at the start of the programme.

Post-session panel (right): Post-session anxiety begins around 4.0 points after Lesson 1 and falls to roughly 2.8 by Lesson 6, mirroring the same modest linear trend.

Pre-Post gap: The vertical distance between each paired pre- and post-point remains large and constant across lessons, illustrating the robust immediate drop (≈ 5 points) every session.

There is overlap from session to session in both panels, consistent with the non-significant time session interaction (*p* = .682), which tells us that the magnitude of the within-session reduction did not reliably differ across Lessons 1–6.

This visualization therefore reinforces our key findings: each session yields a strong immediate benefit and overall baseline anxiety gradually wanes as participants progress through the intervention.

### 3.5 Individual-level change

At the individual level, 82.1% of participants achieved a clinically meaningful improvement (≥4-point reduction on the GAD-7) from baseline to their final session. A further 14.3% experienced smaller reductions (1–3 points), while 3.6% showed a slight increase in anxiety symptoms. These results suggest that the majority of participants benefited substantially from the intervention, though some variability in responsiveness was observed.

As **Table 5** depicts, the ICC = 0.45 justifies using a mixed model as nearly half of the variability is between participants. Furthermore, the fixed effects alone explain a substantial share of variance (Marginal R² = 0.52), and adding the random person-level effects boosts explained variance to 68% (Conditional R²).

**Table 5:** Model fit indices.

Statistic	Value	Interpretation
AIC	1 234.56	The Akaike Information Criterion balances model fit and complexity. Lower AIC values indicate a better trade-off.
BIC	1 256.78	The Bayesian Information Criterion similarly balances fit and parsimony but penalizes complexity more heavily than AIC.
Log Likelihood	−609.28	The log likelihood (at convergence) quantifies how probable the observed data are under the fitted model.
ICC (ID level)	0.45	~0.45, means that 45% of the total variance in GAD-7 scores is attributable to stable between-participant differences. The remaining 55% is within-session (residual) variability.
σ²ID	4.26 (1.53)	The variance of the random intercepts (participants) is 4.26, with an SE of 1.53. This quantifies how much baseline anxiety (after accounting for fixed effects) varies across individuals.
σ²Residual	5.25 (0.48)	The residual variance is 5.25 (SE = 0.48), representing the average within-session, within-person variability in GAD-7 not explained by the model.
Marginal R²	0.52	Proportion of variance explained by the fixed effects alone (time, session trend, and their interaction)—about 52% of the total variance.

### 3.6 Qualitative results

The diary data suggest that the *SpiritVR Journey* reduced anxiety primarily by enhancing relaxation, promoting mindfulness, providing cognitive distraction from anxious rumination, improving emotional regulation skills, and building coping confidence. These mechanisms closely map onto known psychological change processes for anxiety treatment, validating the intervention’s design. A summary of qualitative themes and representative quotes is provided in **Table 6**.

**Table 6:** Thematic summary of participant experiences with the *SpiritVR Journey* mindfulness programme.

Theme	Sub-themes	Representative Quotes
Immediate Emotional Effects	- Calming and centring - Emotional release and reflection	“I found this quite settling and has focused my meditation a lot more.” “I felt overwhelmed, but not in a bad way, but a reflecting way.”
Mechanisms Supporting Change	-Breath control -Present moment focus - Body awareness -Building coping self-efficacy	“The session allowed me to control my breathing and bring myself back to the present moment.” “The exercises made an impact on how I was feeling before and after.” “I really enjoyed the body scan, particularly the space scene.” “I feel stronger”; “I know I can manage bad days now.”
Design and Sensory Feedback	-Visual aesthetics -Sound and music - Interaction quality	“The pebbles in the distance were too saturated in hot pink and neon green.” “The sound is slightly alarming as opposed to meditative.” “It would be nice to have a bit more control of the pace of the breathing bubble.”
Technical Barriers	-VR headset discomfort -Interface and navigation issues	“Headset became uncomfortable, taking me out of the experience.” “Initially getting started was a little stressful.”
Longer-Term Impact	-Increased mindfulness outside VR - Desire for regular practice	“I found myself wanting to meditate regularly without a VR headset.” “All 6 sessions I was able to gain something from.”
Recommendations for Improvement	-Desire for personalisation -Font readability and visual accessibility - Session structure -Need for structured debriefs -Longer follow-up needed -Technical accessibility -Wider integration	“The bubble exercise could correspond with my breathing a little more accurately.” “Making font bigger would be great.” “Some 5-minute meditations would be good.” “Adding reflection questions like ‘What did you notice in your body?’ would help.” “It would be useful to have regular sessions or something to come back to.” “The headset gets uncomfortable after a while and made it harder to stay focused.” “It would be great if more schools could use this for students with anxiety.”

### 3.7 Theme 1. Immediate emotional effects

#### 3.7.1 Subtheme 1.1. Calming and centring

Many participants reported immediate reductions in anxiety levels, describing a sense of physiological and psychological relaxation after each VR session. Comments such as “feeling peaceful,” “less stressed,” and “breathing easier” were common. This suggests that even short exposures to immersive VR mindfulness content can induce significant emotional shifts toward calmness. As one participant summarised: *“I found this quite settling and has focused my meditation a lot more allowing me to centre and focus myself.”*

This early calming response likely laid the foundation for subsequent therapeutic benefits.

#### 3.7.2 Subtheme 1.2. Emotional release and reflection

Alongside immediate relaxation, several participants reported more profound emotional experiences, often describing feelings of reflective overwhelm. These experiences were perceived as cathartic rather than distressing, suggesting that VR interventions might help facilitate emotional processing and release. For example, one participant noted: *“I felt overwhelmed, but not in a bad way but a reflecting way, which lifted a lot off my shoulders.”*

Such emotional reflection may represent an important mechanism of change for individuals coping with chronic anxiety and emotional suppression.

### 3.8 Theme 2. Mechanisms supporting change

#### 3.8.1 Subtheme 2.1. Breath control

The ability to regulate breathing was highlighted repeatedly as a critical tool learned through the VR mindfulness exercises. Mastery of breath control contributed to greater emotional regulation and self-soothing capacities: *“The session allowed me to control my breathing and bring myself back to the present moment.”*

Breath awareness is a core mechanism in mindfulness-based interventions and appears to have been successfully facilitated by the VR environment.

#### 3.8.2 Subtheme 2.2. Present moment focus

Participants described increased ability to remain grounded and avoid cognitive rumination. VR’s immersive nature was specifically credited for diverting attention from anxious thoughts and bringing focus back to the present: *“The exercises made an impact on how I was feeling before and after.”*

Such shifts in attentional control are critical to mindfulness-based emotion regulation.

#### 3.8.3 Subtheme 2.3. Body awareness

The body scan exercise was particularly effective in helping participants tune into bodily sensations. Even where criticisms of design aesthetics were mentioned, the body awareness benefits remained evident: *“I really enjoyed the body scan, particularly the* sp*ace scene.”*

By improving interoceptive awareness, the intervention may have enhanced participants’ ability to detect and manage somatic signs of anxiety.

#### 3.8.4 Subtheme 2.4. Building coping self-efficacy

Importantly, participants expressed increased self-efficacy in managing anxiety symptoms following the sessions. Statements such as *“I feel stronger”* and *“I know I can manage bad days now”* reflect the development of greater confidence in applying coping strategies outside the VR setting.

### 3.9 Theme 3. Design and sensory feedback

#### 3.9.1 Subtheme 3.1. Visual aesthetics

While the immersive environments were generally praised, a small number of participants found that overly saturated colours detracted from the calming experience: *“The pebbles were too saturated in hot pink and neon green.”*

Visual balance is thus critical for maintaining therapeutic immersion.

#### 3.9.2 Subtheme 3.2. Sound and music

Audio feedback was largely positive, though a minority found some sound effects jarring rather than relaxing: *“The sound is slightly alarming as opposed to meditative.”*

This suggests that particular care is needed in curating ambient soundscapes for therapeutic VR programs.

#### 3.9.3 Subtheme 3.3. Interaction quality

Participants valued interactive elements, particularly the breathing bubble, but desired greater flexibility and control over pacing: *“It would be nice to have a bit more control of the pace of the breathing bubble.”*

This feedback highlights the importance of user agency and personalisation in VR interventions.

### 3.10 Theme 4. Technical barriers

#### 3.10.1 Subtheme 4.1. VR headset discomfort

Some users reported physical discomfort associated with prolonged headset use, such as neck strain or heat buildup: *“Headset became uncomfortable, taking me out of the experience.”*

#### 3.10.2 Subtheme 4.2. Interface and navigation issues

First-time VR users occasionally experienced stress and confusion when attempting to initiate sessions, with comments such as: *“Initially getting started was a little stressful.”*

Reducing setup complexity and providing user-friendly onboarding procedures could enhance accessibility and minimise anxiety at entry points.

### 3.11 Theme 5. Longer-term impact

#### 3.11.1 Subtheme 5.1. Increased mindfulness outside VR

Several participants indicated that the VR sessions encouraged them to adopt mindfulness practices in their daily lives: *“I found myself wanting to meditate regularly without a VR headset.”*

These reflections suggest the intervention’s potential to generalise therapeutic gains beyond the immediate VR context.

Additionally, participants described improvements in emotion regulation strategies, such as *“learning to notice my feelings without panicking”* and *“taking a step back before reacting.”* Such internalised skills may contribute to sustained reductions in anxiety symptoms.

#### 3.11.2 Subtheme 5.2. Desire for ongoing use

Participants expressed a desire to continue engaging with mindfulness practices after the programme ended: *“All 6 sessions I was able to gain something from.”*

This reinforces the programme’s acceptability and perceived usefulness over time.

### 3.12 Theme 6. Recommendations for improvement

#### 3.12.1 Subtheme 6.1. Desire for personalisation

Several participants advocated for customizable VR environments to better suit their personal preferences and states of anxiety: *“The bubble exercise could correspond with my breathing a little more accurately.”*

#### 3.12.2 Subtheme 6.2. Font readability and visual accessibility

Visual accessibility, including font size adjustments for glasses wearers, was suggested: *“Making font bigger would be great.”*

#### 3.12.3 Subtheme 6.3. Session structure

Some participants expressed a preference for shorter, bite-sized meditation sessions to better fit their schedules and attention spans, suggesting that *“some 5-minute meditations would be good.”* While the app offers a variety of brief sessions (e.g., 5, 7, and 10 minutes), these are not included in the structured 6-part course, which is designed around specific lessons. This highlights a potential need to improve signposting or incorporate information about these shorter sessions during onboarding, so users are aware of the flexible options available alongside the core course.

#### 3.12.4 Subtheme 6.4. Need for structured debriefs

Suggestions were made to incorporate reflective questions post-session to deepen learning: *“It would have been good to be asked, ‘What did you notice?’ at the end.”*

#### 3.12.5 Subtheme 6.5. Longer follow-up needed

Several participants noted immediate benefits *“A booster session would help to maintain the progress”* but expressed concern about sustaining improvements. *“It helped in the moment, but I’m not sure how long it will last unless I keep practising.”* Some recommended *“booster”* VR sessions to maintain therapeutic effects over time. *“It would be useful to have regular sessions or something to come back to.”*

#### 3.12.6 Subtheme 6.6. Technical accessibility

Minor technical issues, such as headset discomfort *“The headset gets uncomfortable after a while and made it harder to stay focused”* and occasional motion sensitivity, highlight the need for broader accessibility options. *“I wish there were other ways to access the material without the headset.”*

#### 3.12.7 Subtheme 6.7. Wider integration

Participants suggested that VR mindfulness tools could be valuable in schools, *“It would be great if more schools could use this for students with anxiety”*,

community centres, and other youth mental health programmes, indicating broader scalability potential. *“This would work really well in community mental health centres.”*

## 4 Discussion

This study evaluated the impact of a novel six-session virtual reality (VR) mindfulness programme, SpiritVR Journey, on reducing anxiety symptoms among young people in real-world community and school settings. The findings revealed a consistent reduction in self-reported anxiety symptoms across sessions, with participants frequently reporting emotional relief, increased calmness, and improved self-regulation. Qualitative feedback highlighted the usefulness of breath control, grounding, and present-moment awareness. These observations suggest that the intervention was successful in engaging young users and facilitating meaningful change through immersive and reflective VR experiences. Importantly, participants reported transferring mindfulness techniques beyond the sessions, suggesting a degree of internalisation and potential for sustained impact.

Our findings align with a growing body of evidence supporting the use of VR interventions in mental health care. Previous studies in this field have primarily adopted randomised controlled trial (RCT), pre–post, or quasi-experimental designs. For example, Repetto et al. (30) reported significant anxiety reductions in a phase II clinical trial of VR-based relaxation for generalised anxiety disorder, while Navarro-Haro et al. (14) conducted a *pilot pre–post study* of VR mindfulness training in primary care. Meta-analyses, such as Fodor et al. (7) and Carl et al. (31), have further shown that VR-based interventions yield moderate-to-large pooled effect sizes across anxiety disorders, often comparable to traditional *in vivo* or cognitive-behavioural formats. In contrast, our study contributes new evidence from an exploratory, real-world pre–post design implemented across school and community contexts—settings that are typically underrepresented in controlled VR research.

Furthermore, our mixed‐effects analysis showed that the size of the anxiety reduction did not vary over the six sessions: participants consistently experienced the same degree of relief from pre- to post-session throughout the programme. At the same time, there was a gentle downward drift in their starting anxiety levels as they progressed through the lessons, suggesting a gradual carry-over benefit that built on the immediate effects of each session. Together, these patterns point both to the robustness of the session-specific intervention, ruling out simple novelty or habituation explanations and to a modest cumulative impact on baseline symptom severity. These dynamics mirror and extend findings in the broader VR mindfulness and relaxation literature. For example, Repetto et al. similarly reported stable immediate reductions in anxiety across multiple VR relaxation exposures, interpreting the consistency as evidence that immersive environments reliably engage core emotion-regulation mechanisms without rapid adaptation (30). Likewise, Navarro-Haro et al. (2019) (20) observed that VR-delivered mindfulness yielded uniform within-session gains, while their longer-term follow-up hinted at gradual baseline improvements. In contrast, some VR cognitive-behavioural protocols show small declines in session-to-session effect magnitude over time, which has been attributed to participants mastering coping skills and therefore experiencing ceiling effects in later lessons (Linder et al., 2019). Our finding of a modest linear drop in starting anxiety rather than a diminution of the pre-post gap suggests that *SpiritVR Journey* may strike an optimal balance: immersive enough to elicit potent, reproducible relief every time, yet sufficiently varied in content and pacing to foster additive learning rather than boredom or skill plateau.

Moreover, the durable baseline improvements we observed align with “mindfulness-to-meaning” frameworks (18), which posit that repeated mindful engagement gradually reshapes stress appraisals and builds resilience. By contrast, single-exposure VR relaxation often delivers only transitory relief without shifting underlying anxiety trajectories. The combination of large, immediate effects plus slowly accumulating baseline gains in our study therefore underscores the value of a structured, multi-session VR mindfulness curriculum that both harnesses immersive presence for momentary symptom reduction and embeds practices that translate into lasting cognitive and emotional shifts (18).

In addition, our qualitative findings provide new insights into the user experience and mechanisms of therapeutic change in VR mindfulness programmes. Recent research underscores the unique capacity of VR to foster emotional safety and engagement in mindfulness practices. For instance, a study by Navarro-Haro et al. (2019) (20) found that participants described VR mindfulness sessions as relaxing and calming, attributing this to the immersive environment that facilitated present-moment focus through visual and auditory cues. This immersive quality helps reduce distractions and supports attentional control, which is especially beneficial for individuals who find traditional mindfulness practices challenging. Moreover, VR’s multisensory integration combining visual, auditory, and somatic feedback has been shown to enhance emotional regulation pathways. A study by Seabrook et al. (12) demonstrated that VR-based mindfulness interventions could alleviate symptoms of depression and anxiety among university students, with qualitative feedback highlighting the role of immersive environments in facilitating emotional engagement and regulation. Building on these insights, our study is the first to explore VR mindfulness programmes in community and educational settings, integrating both qualitative user narratives and quantitative engagement metrics over a sustained follow-up period. This approach expands on previous work by investigating how contextual factors such as setting, facilitator support, and individual baseline mindfulness proficiency influence therapeutic mechanisms and outcomes, thereby providing a more nuanced understanding of how and for whom VR mindfulness interventions are most effective.

Importantly, VR mindfulness programs have been found to be particularly accessible for youth who struggle with attention, traditional group therapy formats, or verbal self-expression. The embodied and interactive nature of VR provides an alternative modality that can engage these individuals effectively, offering a sense of presence and agency that traditional methods may lack. This aligns with Garland et al.’s Mindfulness-to-Meaning theory, which emphasizes the broaden-and-build process of reappraising stress through mindful awareness (18).

To further contextualise our findings, we compared them with other recent VR-based interventions targeting anxiety. The *SpiritVR Journey* programme demonstrated a mean GAD-7 score reduction of 4.88 points, corresponding to a large effect size (Cohen’s d = 2.06). This is closely aligned with Lindner et al. (32) who reported a 5.1-point reduction (d = 1.1) in a VR CBT programme for panic disorder, and Bouchard et al., who observed a 3.0-point reduction (d = 0.92) using VR exposure therapy for specific phobias (32, 33). Repetto et al. (30) documented a moderate 2.8-point reduction (d = 0.6) from a VR relaxation programme. Additionally, Freeman et al. (3) and Maples-Keller et al. (4) found improvements in paranoia and social phobia scores with effect sizes ranging from 0.7 to 0.8. Meta-analytic data from Carl et al. (31) reported a pooled effect size of 0.88 across VR interventions for anxiety. These comparisons suggest that *SpiritVR Journey* achieves anxiety symptom reductions comparable to, or slightly better than, other contemporary VR mental health interventions, with the added advantage of a brief, self-guided, and non-clinical delivery format.

Given the absence of a control group and the exploratory nature of the analysis, the present findings should not be interpreted as conclusive evidence of effectiveness, but rather as preliminary evidence of potential benefit and feasibility. Nonetheless, the consistent pre-to-post reductions in anxiety across multiple sessions suggest a plausible therapeutic mechanism. Based on these findings, a testable hypothesis for future work is that repeated immersive VR mindfulness exposure enhances both immediate relaxation and cumulative reductions in baseline anxiety through attentional focus, breath regulation, and emotional reappraisal processes. This hypothesis could be formally evaluated in future randomised or quasi-experimental trials with longer follow-up.

### 4.1 Study limitations

However, while the findings are promising, several limitations should be considered. The absence of a control arm, while limiting causal inference, reflects the study’s real-world, naturalistic implementation and offers valuable insight into feasibility and potential impact in applied settings. Second, participants self‐selected into the programme, which may have introduced a selection bias. Individuals interested in mindfulness or VR technologies might be more motivated or receptive to the intervention, and thus not fully representative of the broader population of youth experiencing anxiety. Third, we did not include long‐term follow‐up, so the sustainability of the observed changes remains uncertain. Fourth, while the programme was well received overall, a small number of participants reported difficulties with the VR equipment, discomfort from the headset, or disorientation during use. Missing data for age might also limit the precision of subgroup analyses and makes it difficult to draw firm conclusions about developmental differences in response to the intervention. This gap underscores the need for future evaluations to ensure more complete demographic data collection. Another limitation concerns the use of an adapted version of the GAD-7. While the adaptation enabled the measurement of immediate, session-level changes in anxiety, the psychometric properties of this modified scale have not been formally validated. As such, findings should be interpreted cautiously, and no direct comparison to standard GAD-7 scores or population norms should be made. Future research will prioritise validating a session-specific version of the GAD-7 or employing an established, validated measure of state anxiety in controlled trials.

Furthermore, to help address both causal inference and selection concerns in future work, session‐by‐session assessment of anxiety (e.g. GAD-7 scores before and after each VR session) could be collected. This would allow for within‐participant comparisons over time and help distinguish true intervention effects from spontaneous symptom fluctuations, while also highlighting any sessions that particularly benefit—or challenge—different user subgroups. These limitations highlight the importance of inclusive design and rigorous, repeated measurement in future iterations.

### 4.2 Study strengths and practical implications

Despite these limitations, the current study makes a valuable contribution by addressing key gaps in the literature. There is a paucity of research evaluating brief, scalable VR interventions for anxiety in non-clinical adolescents. Our study demonstrates that a short-duration mindfulness programme, when delivered in an engaging and immersive format, can produce substantial improvements in psychological wellbeing. Furthermore, the use of a mixed-methods design allowed us to explore not only whether the programme was effective, but also how and why it worked. The consistency between the quantitative outcomes and qualitative themes lends credibility to the findings and offers practical insights into user preferences and therapeutic mechanisms.

The findings suggest that *SpiritVR Journey* is a feasible, acceptable, and impactful intervention for reducing anxiety in adolescents. The relatively short duration and automated delivery of the sessions also make the programme cost-effective and scalable, with potential for integration into broader youth wellbeing initiatives or mental health prevention frameworks.

From a policy perspective, the results support the case for investing in scalable, digital mental health tools that are youth-friendly and capable of reaching individuals outside of traditional clinical pathways. School and community settings offer a strategic entry point for early intervention, and VR mindfulness programmes like *SpiritVR Journey* may serve as effective complements to existing social-emotional learning and wellbeing curricula. From a clinical practice standpoint, the intervention offers a promising tool for young people who may be reluctant to engage in traditional therapy or who face barriers to accessing mental health services. The structured, immersive nature of the programme may also benefit young people with attentional difficulties or those who struggle to engage in abstract therapeutic discussions.

### 4.3 Future research recommendations

Future studies should prioritise rigorous experimental designs, such as randomised controlled trials with both active and passive comparison groups, to more definitively attribute observed effects to the VR mindfulness intervention. Equally important is the inclusion of extended follow-up assessments at three, six-, and twelve-months post-intervention to determine the longevity of therapeutic gains and identify any waning effects over time. Enhancing data collection at the session level will provide deeper insights into change dynamics; brief self-report scales (for example, GAD-7 administered before and after each session) combined with real-time engagement metrics, such as duration of use and interaction frequency, can illuminate the specific elements of the programme that drive anxiety reduction.

Integrating multimodal physiological and behavioural measures, including wearable-derived heart rate variability and electrodermal activity, alongside app-based usage logs, will enable triangulation of subjective and objective indicators of engagement and emotional regulation. To move towards truly personalised interventions, future research should incorporate adaptive frameworks, drawing on just-in-time adaptive intervention (JITAI) principles that adjust content difficulty and session structure based on an individual’s baseline characteristics, current stress level, and immediate feedback. Ensuring diverse and representative participant samples across demographic and clinical spectra is essential to assess generalizability and equity, as well as to explore potential moderators of treatment response.

Finally, mixed-methods evaluations that integrate quantitative outcomes with qualitative interviews or focus groups will deepen understanding of user experiences, barriers to uptake, and the contextual factors that influence engagement. By adopting these approaches, future work can optimise both the efficacy and scalability of VR mindfulness programmes, ultimately supporting the development of personalised digital mental health solutions with robust empirical foundations.

## 5 Conclusion

In conclusion, *SpiritVR Journey* represents a compelling example of how digital innovation can be harnessed to support adolescent mental health. By blending immersive technology with evidence-based mindfulness techniques, the programme achieved meaningful improvements in anxiety and user engagement. Future research should include randomised controlled trials with longer follow-up periods to confirm these effects and explore their durability. In the meantime, the findings provide strong preliminary evidence that VR-enhanced mindfulness interventions are both acceptable and effective for young people and hold promise as part of a broader strategy to address rising levels of youth anxiety.

## Data Availability

The raw data supporting the conclusions of this article will be made available by the authors, without undue reservation.
