# Supplementary material for: Evaluating a virtual reality–delivered mindfulness intervention for anxiety: a mixed-methods study in real-world community and school settings
Source: Front Psychiatry. 2025 Dec 11;16:1669287. doi: 10.3389/fpsyt.2025.1669287 (PMC12738872; doi:10.3389/fpsyt.2025.1669287)
Supplement: Supplementary file 1 [file DataSheet1.pdf]

WORKBOOK

Spirit VR

Virtual Reality Well-being

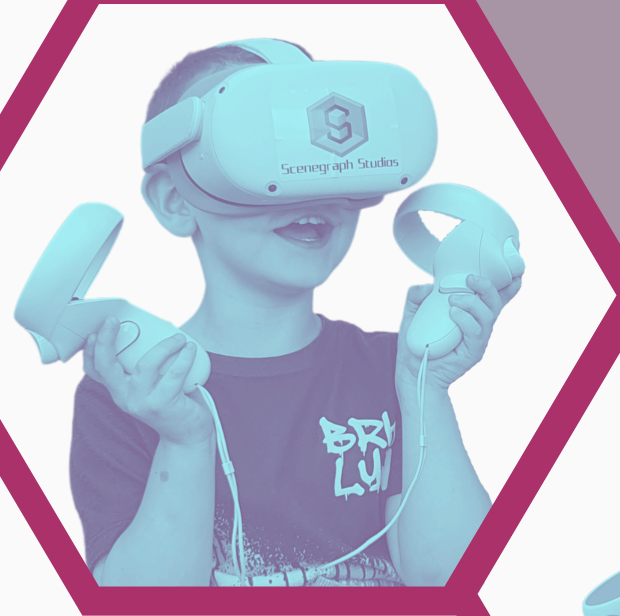

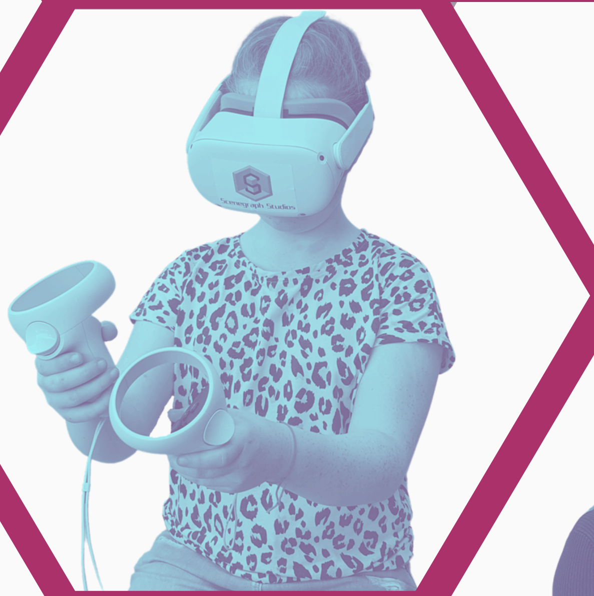

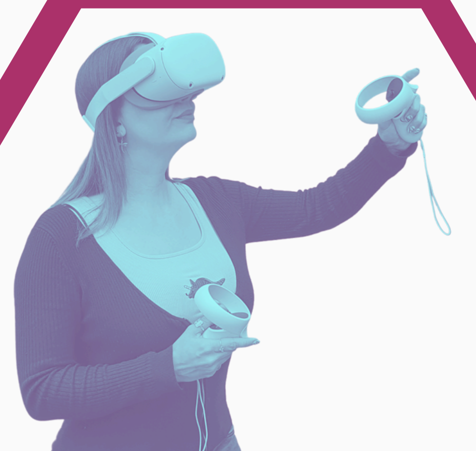

ORCHA CERTIFIED

UKRI Innovate UK

Spirit VR

Virtual Reality Well-being

Thank you for trying SpiritVR – Journey, our VR Mental Well-being application aiming to teach mindful practices within VR. Please visit SpiritVR.co.uk/contact-us to leave any feedback, this will help us greatly and will inform us into how we can make our well-being apps better.

There are six sessions to the course. Ideally, these should be completed with a short break in between each session (2 - 7 days apart).
Each session is designed to teach you a mindful practice. You will be asked to record your mental well-being before and after each session, then use what you have learnt in the days following the session, in your everyday life.

Where can I go for help?

Your doctor (GP) - Your local GP practice is the first place to go to find help you with your mental and physical health.

Samaritans - Freephone 116 123 (24 hours a day, 365 days a year).

National Suicide Prevention Helpline UK - Call 0800 689 5652 (6pm - midnight every day).

Campaign Against Living Miserably (CALM) - Call 0800 58 58 58 (5pm - midnight every day)

UKRI Innovate UK
Funded by Innovate UK, the UK's national innovation agency

ORCHA CERTIFIED
Organisation for the Review of Care and Health Apps

Lesson 1 - Mindful Senses

This session is about taking some time to use our senses to bring us into the present moment. This starts with taking in our surroundings, looking at what we see nearby, then in the distance. Paying close attention to the colours, patterns and shapes we may see. We can also observe to the different sounds that we hear.

Please fill in **Before** starting the session:
(Select one box for each answer)

	Not at all	Mildly	Moderately	Severely
I am feeling nervous, anxious or on edge				
I am not able to stop or control worrying				
I am worrying too much about different things				
I am having trouble relaxing				
I feel so restless that it is hard to sit still				
I feel easily annoyed or irritable				
I feel afraid, as if something awful might happen				

Please fill in **After** completing the session:

	Not at all	Mildly	Moderately	Severely
I am feeling nervous, anxious or on edge				
I am not able to stop or control worrying				
I am worrying too much about different things				
I am having trouble relaxing				
I feel so restless that it is hard to sit still				
I feel easily annoyed or irritable				
I feel afraid, as if something awful might happen				

Additional Activity

You may want to spend some time after this session, in your everyday life, practising mindful senses. This could include spending 10 minutes taking in the environment around you, observing what you see and hear. Then spending a couple of minutes with an everyday item, studying it as if you are seeing and feeling it for the first time.

Lesson 1 - Diary

You may want to use the space below to note any mindful practices that you have applied to your everyday life following this session.

Lesson 2 - Mindful Breathing

This session is about focusing on our breathing to help us feel calmer and more relaxed. Breathing in though the nose slowly and deeply, then out through the nose or mouth slowly and fully, we can calm our feelings of anxiousness.

Please fill in **Before** starting the session:
(Select one box for each answer)

	Not at all	Mildly	Moderately	Severely
I am feeling nervous, anxious or on edge				
I am not able to stop or control worrying				
I am worrying too much about different things				
I am having trouble relaxing				
I feel so restless that it is hard to sit still				
I feel easily annoyed or irritable				
I feel afraid, as if something awful might happen				

Please fill in **After** completing the session:

	Not at all	Mildly	Moderately	Severely
I am feeling nervous, anxious or on edge				
I am not able to stop or control worrying				
I am worrying too much about different things				
I am having trouble relaxing				
I feel so restless that it is hard to sit still				
I feel easily annoyed or irritable				
I feel afraid, as if something awful might happen				

Additional Activity

You may want to spend some time after this session practising mindful breathing. This could include counting your breaths in sets of four, repeating "Breathing in, breathing out, one, breathing in, breathing out, two" until you reach four. Repeat this entire process four times. You may find it helpful to count each set using your hands.

Lesson 2 - Diary

You may want to use the space below to note any mindful practices that you have applied to your everyday life following this session.

Lesson 3 - Body Scan

This session is about noticing how you body feels, without any judgement and without trying to change it in any way. This helps us to reconnect with our bodies, taking us from a place of thinking, to a place of being.

Please fill in **Before** starting the session:
(Select one box for each answer)

	Not at all	Mildly	Moderately	Severely
I am feeling nervous, anxious or on edge				
I am not able to stop or control worrying				
I am worrying too much about different things				
I am having trouble relaxing				
I feel so restless that it is hard to sit still				
I feel easily annoyed or irritable				
I feel afraid, as if something awful might happen				

Please fill in **After** completing the session:

	Not at all	Mildly	Moderately	Severely
I am feeling nervous, anxious or on edge				
I am not able to stop or control worrying				
I am worrying too much about different things				
I am having trouble relaxing				
I feel so restless that it is hard to sit still				
I feel easily annoyed or irritable				
I feel afraid, as if something awful might happen				

Additional Activity

You may want to spend some time after this session practising a body scan. When you have some spare time, sit and relax. Start from your feet and work your way up to your head, noticing how your body feels, without trying to change it in any way.

Lesson 3 - Diary

You may want to use the space below to note any mindful practices that you have applied to your everyday life following this session.

Lesson 4 - Mindful Thoughts

This session is about concentrating on our thoughts and feelings as they happen in the moment. Allowing our thoughts to take centre stage, acknowledging them without judgement, then allowing them to pass us by.

Please fill in **Before** starting the session:
(Select one box for each answer)

	Not at all	Mildly	Moderately	Severely
I am feeling nervous, anxious or on edge				
I am not able to stop or control worrying				
I am worrying too much about different things				
I am having trouble relaxing				
I feel so restless that it is hard to sit still				
I feel easily annoyed or irritable				
I feel afraid, as if something awful might happen				

Please fill in **After** completing the session:

	Not at all	Mildly	Moderately	Severely
I am feeling nervous, anxious or on edge				
I am not able to stop or control worrying				
I am worrying too much about different things				
I am having trouble relaxing				
I feel so restless that it is hard to sit still				
I feel easily annoyed or irritable				
I feel afraid, as if something awful might happen				

Additional Activity

You may want to spend some time after this session thinking about your thoughts. Let a thought enter your mind, acknowledge it, then let it pass by. Remembering, that a thought is just a thought, it doesn't hold power over you and you don't have to agree with it. Try this with five thoughts.

Lesson 4 - Diary

You may want to use the space below to note any mindful practices that you have applied to your everyday life following this session.

Lesson 5 - Mountain Meditation

This session is about envisioning ourselves as a strong, powerful mountain. Watch along as the mountain remains constant and withstands the changes in its environment, remaining unmoved and unaffected throughout.

Please fill in **Before** starting the session:
(Select one box for each answer)

	Not at all	Mildly	Moderately	Severely
I am feeling nervous, anxious or on edge				
I am not able to stop or control worrying				
I am worrying too much about different things				
I am having trouble relaxing				
I feel so restless that it is hard to sit still				
I feel easily annoyed or irritable				
I feel afraid, as if something awful might happen				

Please fill in **After** completing the session:

	Not at all	Mildly	Moderately	Severely
I am feeling nervous, anxious or on edge				
I am not able to stop or control worrying				
I am worrying too much about different things				
I am having trouble relaxing				
I feel so restless that it is hard to sit still				
I feel easily annoyed or irritable				
I feel afraid, as if something awful might happen				

Additional Activity

You may want to spend some time after this session imagining yourself as the mountain if you are feeling overwhelmed with your surroundings. Imagine the weather, the seasons and time unfolding around you, whilst you remain calm, strong and unmoved throughout.

Lesson 5 - Diary

You may want to use the space below to note any mindful practices that you have applied to your everyday life following this session.

Lesson 6 - Loving Kindness Meditation

Now that we have practised mindfulness, allowing us to reflect on our own well-being, we are going to practise kindness. The Loving Kindness Meditation allows us to be more empathetic and compassionate, focusing on the well-being of others.

Please fill in **Before** starting the session:
(Select one box for each answer)

	Not at all	Mildly	Moderately	Severely
I am feeling nervous, anxious or on edge				
I am not able to stop or control worrying				
I am worrying too much about different things				
I am having trouble relaxing				
I feel so restless that it is hard to sit still				
I feel easily annoyed or irritable				
I feel afraid, as if something awful might happen				

Please fill in **After** completing the session:

	Not at all	Mildly	Moderately	Severely
I am feeling nervous, anxious or on edge				
I am not able to stop or control worrying				
I am worrying too much about different things				
I am having trouble relaxing				
I feel so restless that it is hard to sit still				
I feel easily annoyed or irritable				
I feel afraid, as if something awful might happen				

Additional Activity

You may want to spend some time after this session imagining a variety of people in your life, some you may like, others you may struggle to get along with. See if you can offer everyone the same loving kindness by repeating the following as you think of them:
"May you be happy, may you be healthy, may you have the power to forgive and accept, may you live in peace."

Lesson 6 - Diary

You may want to use the space below to note any mindful practices that you have applied to your everyday life following this session.

Spirit
VR

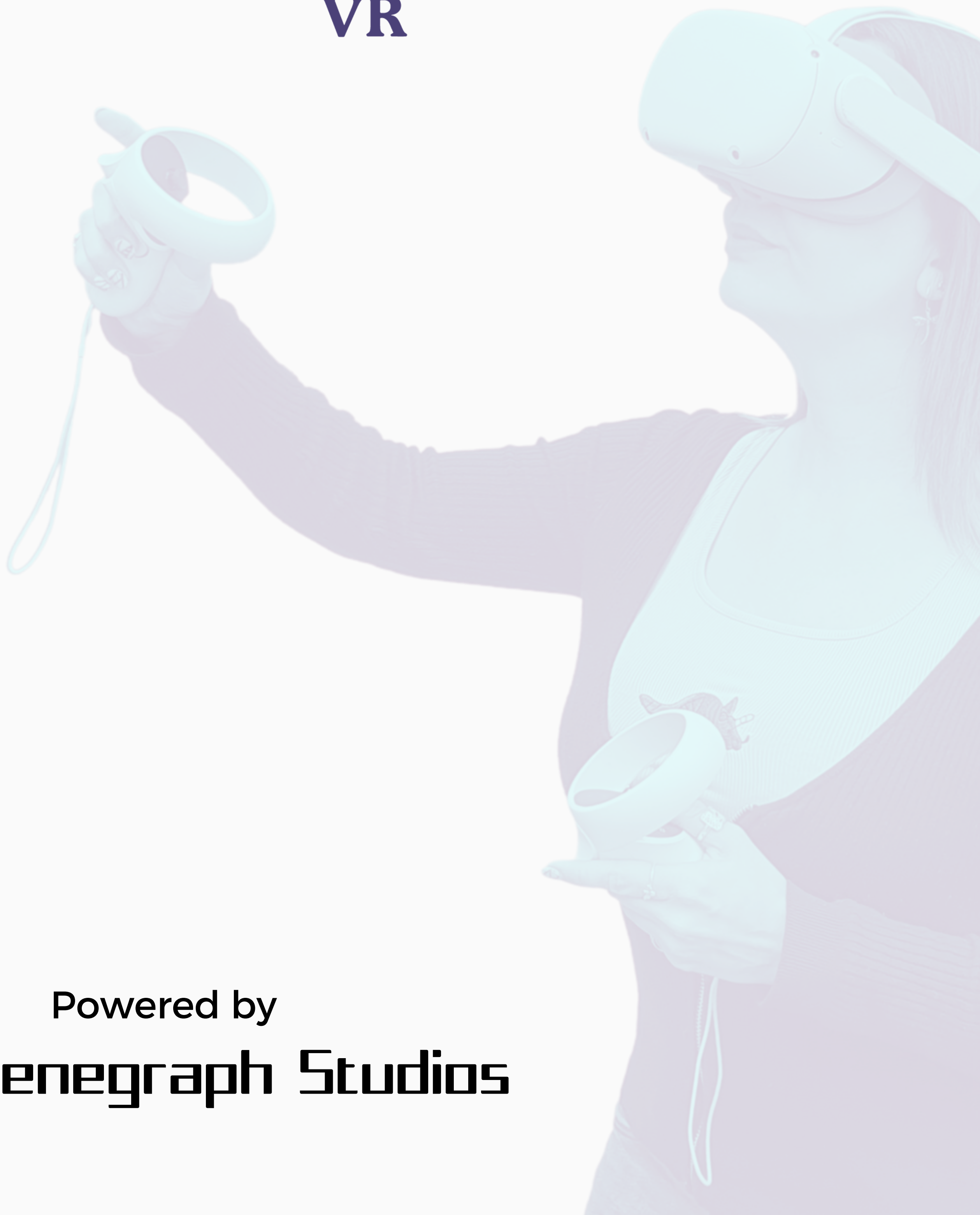

Powered by

Scenegraph Studios
