# Supplementary material for: Evaluating a virtual reality–delivered mindfulness intervention for anxiety: a mixed-methods study in real-world community and school settings
Source: Front Psychiatry. 2025 Dec 11;16:1669287. doi: 10.3389/fpsyt.2025.1669287 (PMC12738872; doi:10.3389/fpsyt.2025.1669287)
Supplement: Supplementary file 2 [file DataSheet2.pdf]

Lesson 1 - Mindful Senses

This session is about taking some time to use our senses to bring us into the present moment. This starts with taking in our surroundings, looking at what we see nearby, then in the distance. Paying close attention to the colours, patterns and shapes we may see. We can also observe to the different sounds that we hear.

Please fill in **Before** starting the session:
(Select one box for each answer)

	Not at all	Mildly	Moderately	Severely
I am feeling nervous, anxious or on edge				
I am not able to stop or control worrying				
I am worrying too much about different things				
I am having trouble relaxing				
I feel so restless that it is hard to sit still				
I feel easily annoyed or irritable				
I feel afraid, as if something awful might happen				

Please fill in **After** completing the session:

	Not at all	Mildly	Moderately	Severely
I am feeling nervous, anxious or on edge				
I am not able to stop or control worrying				
I am worrying too much about different things				
I am having trouble relaxing				
I feel so restless that it is hard to sit still				
I feel easily annoyed or irritable				
I feel afraid, as if something awful might happen				

Additional Activity

You may want to spend some time after this session, in your everyday life, practising mindful senses. This could include spending 10 minutes taking in the environment around you, observing what you see and hear. Then spending a couple of minutes with an everyday item, studying it as if you are seeing and feeling it for the first time.
